# Three brothers with a nonsense mutation in *KAT6A* caused by parental germline mosaicism

**DOI:** 10.1038/hgv.2017.45

**Published:** 2017-11-09

**Authors:** Chisei Satoh, Ryuta Maekawa, Akira Kinoshita, Hiroyuki Mishima, Michiko Doi, Mutsuko Miyazaki, Masafumi Fukuda, Haruo Takahashi, Tatsuro Kondoh, Koh-ichiro Yoshiura

**Affiliations:** 1 Department of Otolaryngology-Head and Neck Surgery, Unit of Translation Medicine, Nagasaki University Graduate School of Biomedical Sciences, Nagasaki, Japan; 2 Department of Human Genetics, Nagasaki University Graduate School of Biomedical Sciences, Nagasaki, Japan; 3 Department of Pediatrics, Nagasaki University Graduate School of Biomedical Sciences, Nagasaki, Japan; 4 Department of Pediatrics, Nagasaki Prefectural Center for Handicapped Children, Isahaya, Japan; 5 Division of Developmental Disability, Misakaenosono Mutsumi Developmental, Medical and Welfare Center, Isahaya, Japan

**Keywords:** Genetic counselling, Neurodevelopmental disorders, Mutation

## Abstract

Mutations in *KAT6A*, encoding a member of the MYST family of histone acetyl-transferases, were recently reported in patients with a neurodevelopmental disorder (OMIM: #616268, autosomal dominant mental retardation-32). In this report, we describe three siblings with intellectual disability (ID) or global developmental delay and a *KAT6A* heterozygous nonsense mutation, i.e., c.3070C>T (p.R1024*, ENST00000406337; chr8:41795056G>A on hg19). This mutation was identified by whole-exome sequencing of all three siblings but not in a healthy sibling. The mutation was not detected in the peripheral blood of their parents, suggesting the existence of parental germline mosaicism. The primary symptoms of our patients included severe to profound ID or global developmental delay, including speech delay with craniofacial dysmorphism; these symptoms are consistent with symptoms previously described for patients with *KAT6A* mutations. Although several features are common among patients with *KAT6A* mutations, the features are relatively nonspecific, making it difficult to establish a clinical entity based on clinical findings alone. To the best of our knowledge, this is the first report of cases with a *KAT6A* mutation in an Asian population and these cases represent the first reported instances of germline mosaicism of this disease.

## Intellectual disability: Novel inheritance of mutation

A rare intellectual disability can be inherited from a mutation found in a parent’s reproductive cells but not other body cells. Koh-ichiro Yoshiura of Nagasaki University and colleagues in Japan analyzed the genes of a family with three siblings affected by intellectual disability. Peripheral blood samples showed the KAT6A gene was mutated in the affected children but not in their healthy sibling or parents. The similar clinical presentations of the affected children suggests inheritance. Absence of the mutation in the parent’s blood indicates it came from a parent whose reproductive or ‘germ’ cells have a different genetic makeup from their body cells, known as ‘germline mosacism’. This is the first reported case of inheritance of this mutation from germline mosaicism and has implications for genetic counseling.

## Introduction

Intellectual disability (ID) is defined as an intelligence quotient of 70 or below and affects ~1% of children worldwide.^1^ ID is caused by environmental or genetic factors, but the explicit cause is not identified in up to 60% of cases.^2^ Approximately 25–50% of ID cases are thought to have a genetic cause.^2^ A recent large-scale sequencing study identified probable pathogenic mutations in ~40% individuals with ID who underwent whole-exome sequencing (WES) or genome sequencing.^3^

WES is a powerful tool in identifying genetic alterations in putative genetic disorders, even those that are undiagnosed, providing a molecular diagnosis rate of 25%.^4^ In addition, WES has revealed that ~8% of patients without a definitive causative mutation harbor novel candidate mutations.^5^ Moreover, for some cases of undiagnosed rare diseases, a new entity has been established among patients whose disorder was previously indistinguishable from other diseases expressing similar phenotypes. Recent studies reported patients with *de novo KAT6A* mutations among individuals diagnosed with known rare autosomal dominant diseases.^6–8^ Although those patients had similar phenotypes, the features were nonspecific. Thus, it is difficult to distinguish this disease from other diseases based on clinical findings alone.

In this report, we describe three siblings with ID or global developmental delay with a *KAT6A* heterozygous nonsense mutation that was potentially transmitted from one of their parents as a germline mosaicism. This result reinforces ‘germline mosaicism’ in genetic counseling for patients with *de novo* mutation.

## Materials and methods

### Subjects

**Figure 1:**
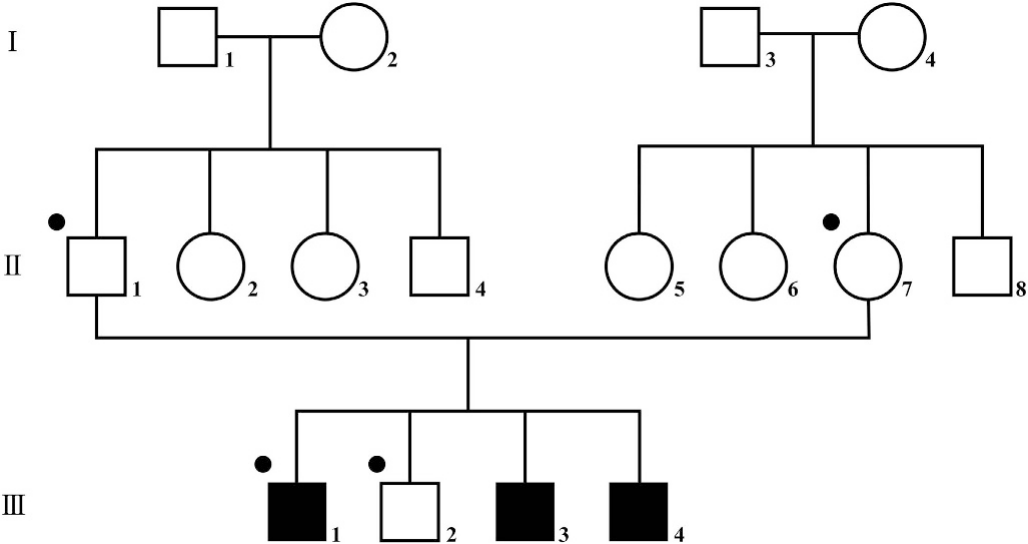
Family pedigree. Three brothers from non-consanguineous parents are affected with the disease. Individuals who underwent whole exome sequencing (WES) analysis are indicated by dots.

Three Japanese brothers from nonconsanguineous parents were analyzed (Figure 1). The brothers exhibited ID and several common features, suggesting that the underlying cause was genetic. The three siblings, their unaffected brother, and their parents were subjected to genetic testing as part of the Initiative on Rare Undiagnosed Diseases in Pediatrics project in Japan. This study protocol has been approved by the Committee for Ethical Issues on Human Genome and Gene Analysis at Nagasaki University. All genetic analyses were performed in the Department of Human Genetics at Nagasaki University.

### Whole-exome sequencing

Peripheral blood was obtained with written informed consent and DNA was extracted using the QIAamp DNA Maxi kit (QIAGEN, Hilden, Germany) according to the manufacturer’s protocol. Four subjects, the parents (II-1 and II-7), an affected child (III-1), and an unaffected child (III-2), underwent WES to screen for the candidate causative mutation (Figure 1). Coding exons were captured with the SureSelect XT AUTO HUMAN ALL Exon V5 kit (Agilent Technology, Santa Clara, CA, USA) and sequenced on a HiSeq2500 system (Illumina, San Diego, CA, USA) in rapid mode with 101 bp paired-end reads. Reads were aligned to GRCh37/hg19 with Novoalign (Novocraft Technologies, Selangor, Malaysia) and duplicate reads that were excluded from the following analysis were marked with Novosort software (Novocraft Technologies). Local realignment and variant calling were performed by the Genome Analysis Toolkit.^9^ Generated variant call format files were processed in the following two manners. First, trio-based VCS format files were filtered to extract the *de novo*, homozygous, and X-linked mutations followed by annotation with ANNOVAR.^10^ Second, variant call format files were annotated with ANNOVAR then filtered to extract compound heterozygous mutations. This process excluded variants with allele frequencies >0.5% in the Exome Aggregation Consortium (http://exac.broadinstitute.org/), NHLBI GO Exome Sequencing Project (http://evs.gs.washington.edu/EVS/), Human Genetic Variation Database (http://www.hgvd.genome.med.kyoto-u.ac.jp), or the database of Tohoku Medical Megabank (http://www.dist.megabank.tohoku.ac.jp). Three *de novo* mutations identified in WES were amplified by PCR in all family members (II-1, II-7, III-1, III-2, III-3, and III-4) followed by direct sequencing to confirm the existence and linkage to phenotype. The primers designed by Primer3Plus (http://www.bioinformatics.nl/primer3plus) were as follows: KAT6A_F, 5′- ATCTCAAACGTGGGTTCTAA-3′, KAT6A_R, 5′- ATGTGCTAATTCTATTTGGT-3′; CEP89_F, 5′- AACTGGGAACATAGAAAACA-3′, CEP89_R, 5′- CAGTGTCAAGTGTTAAGTGA-3′; and CDX4_F, 5′- TCCAATTTCGCTGCGGCACC-3′, CDX4_R, 5′- AGGGCCCAAGTTGCTGTAGTC-3′.

## Results

### Clinical features

Individual III-1 was a 14-year-old boy who was born at 40 weeks of gestation with a birth weight of 2,914 g. He was intubated for breathing problems soon after birth due to persistent pulmonary hypertension and was hospitalized for 2 months. He sat unassisted at 12 months and walked at 22 months of age. He spoke at the age of 3, but his vocabulary was limited to a few short nursery words. He underwent surgery for undescended testes and inguinal hernia at 10 months of age. At the age of 7, his height was 115.2 cm (−0.63 standard deviation (SD)), his weight was 18.8 kg (−0.89 SD), and his head circumference was 49.6 cm (−1.47 SD). His intelligence quotient was 13 at 13 years of age.

Individual III-3 was a 10-year-old boy who was born at 37 weeks of gestation with a birth weight of 2,910 g. He had meconium aspiration syndrome and stayed in an incubator for 2 weeks. He was repeatedly admitted to hospital with recurrent pulmonary infection during his first year of life. He sat unassisted at 18 months, walked at 30 months old, and spoke at the age of 5. At 2 years of age, his height was 79 cm (−1.8 SD), his weight was 7.63 kg (−3.25 SD), and his head circumference was 45 cm (−1.72 SD). His intelligence quotient was 26 at 10 years of age.

Individual III-4 was a 10-month-old boy who was born at 35 months of gestation with a birth weight of 2,270 g. He had an atrial septal defect and mild laryngomalacia. He cannot sit without assistance.

All three affected siblings have the clinical triad characteristic of the family involving mild to moderate scaphocephaly, micrognathia, and low-set ears. In addition, feeding difficulties and axial hypotonia are also observed. Individuals III-1 and III-3 have strabismus (exotropia) and abnormal teeth (malocclusions). Their blood cell counts and serum levels of liver enzymes, creatinine, and electrolytes are within normal ranges. Computed tomography scanning revealed no evidence of intracranial abnormalities.

### WES and Sanger sequencing

**Figure 2:**
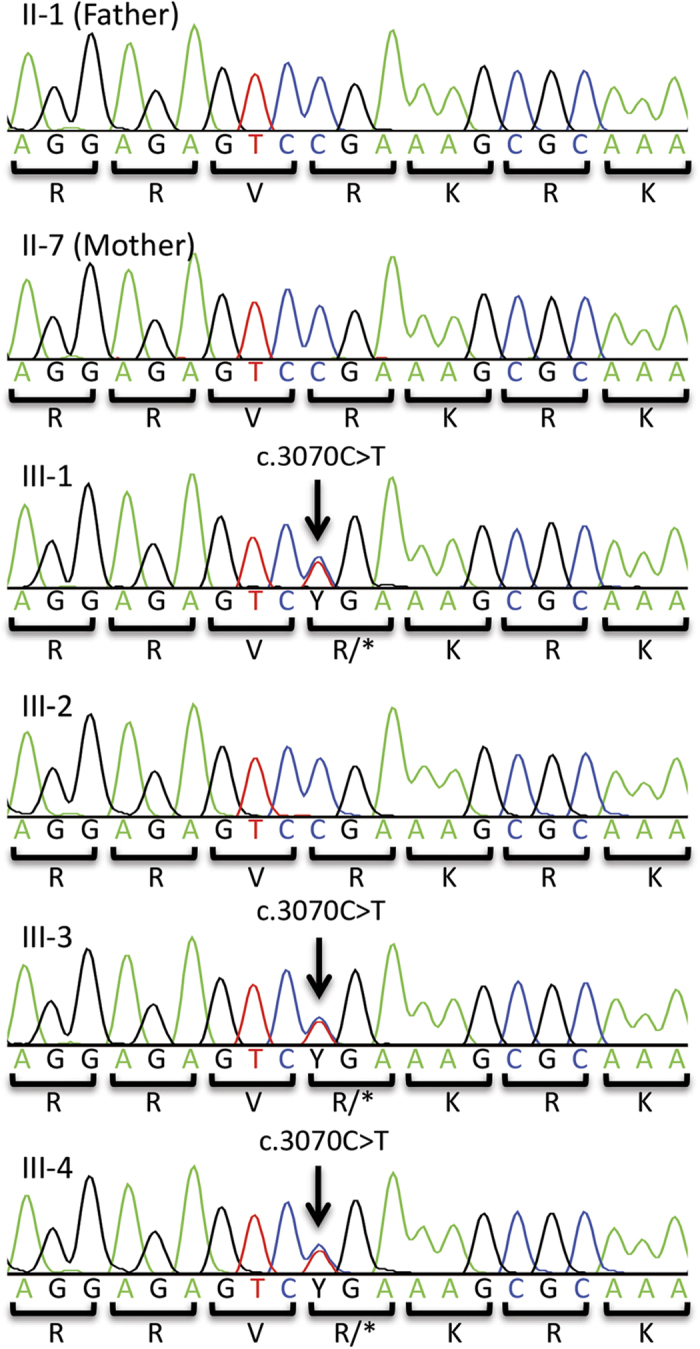
Electropherogram of the mutation locus in this family. The three affected siblings carry the *de novo* heterozygous nonsense mutation (ENST00000406337, c.3070C>T, p.R1024*). This mutation was not detected in the peripheral blood of their parents.

The mean depth of coverage for each individual in WES is as follows: II-1, 74×; II-7, 104×; III-1, 74×; III-2, 77×. Trio analysis revealed that three genes, *KAT6A*, *CEP89*, and *CDX4*, have *de novo* heterozygous single nucleotide variations in the affected individual III-1 but not in unaffected III-2. Sanger sequencing of all family members (II-1, II-7, III-1, III-2, III-3, and III-4) detected the *KAT6A* mutation in affected boys but not in the unaffected boy or the parents. This *de novo* nonsense mutation, c.3070C>T (p.R1024*, ENST00000406337; chr8:41795056G>A on hg19) (Figure 2), was previously reported in two patients.^7,8^ The phenotypes of our cases are similar to those cases, and we concluded that this mutation is causative of disease in the present family (OMIM: 616268) (Table 1).

**Table 1:** Major characteristics of patients with c.3070C>T

	*III-1*	*III-3*	*III-4*	*Arboleda* *et al.* ^7^	*Millan* *et al.* ^8^
Age	14 Years	11 Years	1 Years 3 months	5 Years	5 Years
Gender	Male	Male	Male	Male	Female
Gastational age	40 Weeks	37 Weeks	35 Weeks	42weeks	—
Weight at birth	2,914 g	2,910 g	2,270 g	1,870 g	—
Probrem at birth	Persistent pulmonary hypertention	Meconium aspiration syndrome	Respiratory problems	—	—
*Development*					
Sit unussisted	1 Year	1 Year 6 months	Not yet	1 Year	—
Walk	1 Year 10 months	2 Years 6 months	Not yet	4 Years 6 months	1 Year 7 months
Speach	3 Years (2 words)	5 Years	Not yet	Absent speech	5 Years (3 words)
Intelligence quotient	13 (at 13 years)	26 (at 10 years)	NE	—	—
Measurements (age at evaluation)	(at 6 years 8 months)	(at 2 years 10 months)	(at 1 year 3 months)	(at 4 years 6 months)	
Tall (SD)	−1.6	−1.8	−0.9	−2.6	>75th Percentile
Weight (SD)	−0.89	−3.25	−2.0	−0.7	>75th Percentile
OFC (SD)	−1.47	−1.72	−1.0	−2.52	20–70th Percentile
*Physical features*					
Head	Scaphocephary	Scaphocephary	Scaphocephary	—	—
Face	Micrognathia, low set ear, malocclusions	Micrognathia, low set ear, malocclusions	Micrognathia, low set ear	Lower teeth are smoll and peg-shaped	Epicanthal fold, bulbous nasal tip, micrognathia
Musculoskeletal	Axial hypotonia	Axial hypotonia	Axial hypotonia	Axial hypotonia	—
Eye	Exotropias, myopic astigmatism	Exotropia	NE	Strabismus	Ptosis
Cardiac	NE	NE	NE	NE	Patent ductus atresia
Other feature	Feeding difficulty, orthostatic distention, allergic rhinitis	Feeding difficulty, mild hearing loss	Feeding difficulty, laryngomalacia, curly hair	—	Laryngomalacia

Abbreviations: NE, not evaluated; OFC, occipito-frontal circumference.

## Discussion

In this report, we describe three siblings with ID or global developmental delay caused by a *KAT6A* mutation. To date, 17 cases with ID or global developmental delay from 16 families with *KAT6A* mutations have been reported.^6–8^ A recent large-scale study including more than 7,000 individuals with neurodevelopmental disorder identified 11 patients with potentially damaging *KAT6A de novo* mutations.^3^ This finding may reflect the actual frequency of the disease.

The main symptoms in our cases involved ID and developmental delay with very limited verbal development, which is consistent with previous reports of patients with *KAT6A* mutations. Craniofacial abnormalities, especially a small head circumference (some cases met the criteria of microcephaly), are noted at a high frequency and are relatively characteristic.^6–8^ Low-set ears were observed in all our patients and in 5/7 cases reported by Tham *et al.*^6^ Feeding difficulties and axial hypotonia were also observed in most patients, and many patients had congenital heart disease and strabismus. Scaphocephaly was common among our patients but has not previously been observed in other reported cases. Teeth abnormalities and micrognathia are common in patients with the *KAT6A* p.R1024* mutation. Although several common features can be recognized in individuals with *KAT6A* mutations, these findings are nonspecific, making the disorder indistinguishable from similar diseases with ID.

Analysis of parental genotypes in the present study indicated that the *KAT6A* mutation of our patients is *de novo* but that it must have been transmitted by parental germline mosaicism given its consecutive occurrence. Germline mosaicism refers to variation in the genomes of germline cells within an individual.^11^ Although it can be beneficial to define the mosaic rate of gametes in such cases, we did not do so, because it is practically impossible to use ova for research purposes. Recent deep sequencing or droplet digital PCR techniques are used to detect a very low prevalence of somatic mosaicism in the blood;^12,13^ however, this technology is not useful to determine the germline mosaic rate. Acuna-Hidalgo *et al.*^13^ reported that the genome-wide analysis of putative *de novo* mutations in the proband detected 4/4,081 variants in the blood of one of the parents. In our exome analysis, the mutation was not detected within a read depth of 89 in the father (II-1) and 113 in the mother (II-7).

*KAT6A*, which is also known as *MOZ* or *MYST3* on chromosome 8q11.21, encodes a member of the MYST family of histone acetyl-transferases consisting of 2004 amino acids. *KAT6A* was first described as a fusion protein in patients with acute myeloid leukemia^14^ and all members of this family have a histone acetyl-transferase domain that acetylates the histone lysine residue and promotes transcription.^15^ *KAT6A* is involved in development through the regulation of Hox gene expression.^16^ In zebrafish, the *KAT6A* ortholog specifies segmental identity in the pharyngeal arches.^17,18^ Kastumoto *et al.*^19^ described the *Kat6a* homozygous knockout mouse model, in which exon 2 containing the first ATG of *Kat6a* was replaced with a neo gene cassette, resulting in embryonic lethality and a lack of hematopoietic stem cells. Another homozygous knockout model, in which the neo coding sequence was inserted into exon 16, was also lethal, exhibiting craniofacial and heart abnormalities with normal hematopoietic cells.^16,20^ Although no description exists for heterozygous knockout mouse model defects, the craniofacial and heart abnormalities commonly identified in patients with *KAT6A* could correspond to this model, except for hematopoietic cell aberrations.

In conclusion, we report three siblings with a *KAT6A* mutation and ID. This is the first report of *KAT6A* mutations in an Asian population, and this report also describes the first case of germline mosaicism of the disease. To date, this disease can only be diagnosed after genome-wide testing and the observation of particular craniofacial characteristics. However, the widespread use of WES will lead to an accumulation of patient clinical data that could provide useful information about the diagnosis, prognosis, and future strategies for treating the disease.

## Additional information

**Publisher’s note:** Springer Nature remains neutral with regard to jurisdictional claims in published maps and institutional affiliations.
